# Supplementary material for: Substrate promiscuity of inositol 1,4,5-trisphosphate kinase driven by structurally-modified ligands and active site plasticity
Source: Nat Commun. 2024 Feb 19;15:1502. doi: 10.1038/s41467-024-45917-5 (PMC10876669; doi:10.1038/s41467-024-45917-5)
Supplement: Supplementary file 1 — Supplementary Information [file 41467_2024_45917_MOESM1_ESM.pdf]

Supplementary Information for

Substrate promiscuity of inositol 1,4,5-trisphosphate kinase driven by structurally-modified ligands and active site plasticity

María Ángeles Márquez-Moñino *et al.*

*Corresponding authors. Email: barry.potter@pharm.ox.ac.uk and xbeatriz@iqf.csic.es

Supplementary Methods

The following describes the synthesis of all intermediate steps in a single route to achieve compound **16** based upon[1] (Supplementary Fig. 2):

Synthesis of DL-(1,3,5/2,4,6)-1,3-Di-*O*-benzyl-5,7-*O*-benzylidene-6-hydroxymethyl-2,4-di-*O*-(4-methoxybenzyl)cyclo-hexane 1,2,3,4,5-pentol (16)

1,3,5-*O*-Methylidyne-*myo*-inositol (**22**)
To *myo*-inositol (20.0 g, 0.11 mol) and PTSA (4.7 g, 0.025 mol) in DMF (200 mL) was added triethyl orthoformate (35 mL, 0.210 mol) slowly at 140 °C. The mixture was stirred for 3h, allowed to cool and the DMF was evaporated. 10% Aqueous Na_2CO_3 solution (50 mL) was added. The mixture was stirred for 15 min, filtered then diluted with water (200 mL), the solution was washed with $CHCl_3$ (3 × 100 mL) and the water was evaporated. The residue, in MeOH (600 mL), was heated to 50°C and the remaining solid was filtered off. Evaporation, chromatography (acetonitrile) and recrystallization (MeOH) gave 1,3,5-*O*-methylidyne-*myo*-inositol (**22**) (12.1 g, 57%) as colorless crystals: mp: >275°C (decomposes) (lit. in[1] 300-302°C sealed tube) ; 1H NMR (270 MHz, $[^2H]_6$-DMSO): δ 3.94-3.96 (m, H-1 and H-3, 2H), 3.99 (br s, H-2, 1H), 4.06 (m, H-5, 1H), 4.26-4.29 (m, H-4 and H-6, 2H), 5.33 (br s, OH-2, 1H), 5.45 (s, O_3CH, 1H) 5.47 (br s, OH-4 and OH-6, 2H); analysis (calcd., found for $C_7H_{10}O_6$): C (44.22, 44.3), H (5.30, 5.3).

4,6-Di-*O*-(4-methoxybenzyl)-*myo*-inositol 1,3,5-orthoformate (**23**)
To *myo*-inositol orthoformate (**22**) (11.68 g, 61.5 mmol) in dry DMF (250 mL) was added NaH (60% dispersion in mineral oil, 5.4 g, 141 mmol) slowly at room temperature. The mixture was stirred for 1h then 4-methoxybenzyl chloride (17.5 mL, 129 mmol) was added. After 3h water (50 mL) was added slowly, then the solvents were evaporated. Water (300 mL) was added to the residue and the products were extracted with CH_2Cl_2 (3× 200 mL). The pooled organic phases were washed with brine and was then dried with $MgSO_4$. Evaporation, chromatography (CH_2Cl_2 EtOAc 5:1) and recrystallization (EtOAc/hexane) gave the title compound (10.26 g, 38%) as colorless crystals: mp: 120-121°C (lit.[1] 120-121°C); 1H NMR (270 MHz, $CDCl_3$): δ 3.25 (d, OH-2, J = 11.4 Hz, 1H), 3.78 (s, OCH_3, 6H), 4.13-4.15 (m, H-2, 1H), 4.17-4.21 (m, H-1 and H-3, 2H), 4.33 (dd, H-4 and H-6, J = 3.5 Hz, 2H), 4.39-4.41 (m, H-5, 1H), 4.48 (d, CH_2, J = 11.0 Hz, 2H), 4.56 (d, CH_2, J = 11.0 Hz, 2H), 5.46 (s, O_3CH, 1H), 6.69-6.83 (m, Ar-H, 4H), 7.15-7.19 (m, Ar-H, 4H); 13C NMR (68 MHz, $CDCl_3$,): δ 55.14 (OCH_3), 61.35 (Ins C), 67.77 (Ins C), 71.26 (CH_2), 72.95 (Ins C), 73.40 (Ins C), 103.21 (O_3C), 113.78 (Ar C), 129.30 (Ar C), 129.54 (Ar C), 159.32 ($COCH_3$); analysis (calcd., found for $C_{23}H_{26}O_8$): C (64.18, 6.1), H (6.09, 6.1).

2,4,6/3,5-Pentahydroxy-3,5-di-*O*-(4-methoxybenzyl)-2,4,6-*O*-methylidyne-cyclohexanone (**12**)
To oxalyl chloride (12.8 mL of a 2.0 M solution in CH_2Cl_2, 25.6 mmol) in dry CH_2Cl_2 (40 mL) was added dry DMSO (3.6 mL, 51 mmol) in dry CH_2Cl_2 (5 mL) dropwise at -60°C under N_2. The mixture was stirred for 10 min at -60°C. The alcohol **23** (10.00 g, 23.2 mmol) in dry CH_2Cl_2 (30 mL) was added dropwise. The mixture was stirred for 30 min at -60°C, then triethylamine (15 mL) was added. The mixture was allowed to warm to room temperature, then water (100 mL) was added and the product extracted with CH_2Cl_2 (2 × 150 mL). The organic layer was washed with brine (200 mL), 1% hydrochloric acid (150 mL), water (150 mL), 10% aqueous $NaHCO_3$ solution (200 mL), and water (200 mL), then dried with $MgSO_4$. Evaporation gave a white solid which was dissolved in toluene (300 mL) and heated to reflux with azeotropic removal of water using a Dean and Stark trap for 3h. Evaporation then recrystallization (EtOAc/hexane) under N_2 gave the ketone **12** (8.13 g, 82%) as colorless crystals: mp: 126-127°C (lit.[1]125-126°C); IR (Nujol) 1760cm^{-1} CO; 1H NMR (400 MHz, $CDCl_3$): δ 3.78 (s, OCH_3, 6H), 4.39 (dd, H-3 and H-5, J = 2.5 Hz, 1.5 Hz, 2H), 4.48-4.50 (m, H-4, 1H), 4.51-4.54 (m, CH_2, H-2 and H-6, 6H), 5.63 (s, O_3CH, 1H), 6.80-6.84 (m, Ar-H, 4H), 7.14-7.18 (m, Ar-H, 4H); 13C NMR (100 MHz, $CDCl_3$): δ55.23 (CH_3), 68.87 (Ins C), 71.17 (CH_2) 76.35 and 77.98 (Ins C), 102.63 (O_3CH), 113.82 and 129.51 (Ar C), 128.96 (*C*CH_2), 159.45 (Ar*C*OCH_3), 199.28 (CO).

2,4-di-*O*-(4-methoxybenzyl)-6-methylene-1,3,5-*O*-methylidyne-cyclohexane 1,3,5/2,4-pentol (**24**)
To methyltriphenylphosphonium bromide (12.4 g, 34.7 mmol) in dry THF (40 mL) was added potassium *tert*-butoxide (33 mL of a 1 M solution in THF, 33 mmol) at 0 °C under N_2, with stirring. The yellow suspension was allowed to reach room temperature. After 10 min, the ketone **12** (7.01 g, 16.4 mmol) in dry THF (60 mL) was added. The mixture was heated to reflux for 3h. The THF was evaporated and the residue was dissolved in diethyl ether (200 mL), washed with brine (200 mL) then dried with $MgSO_4$. Evaporation, then chromatography (EtOAc/ hexane 1:2) gave the alkene **24** (5.89 g, 84%) as a white solid: mp: 90-91°C (from EtOH) (lit.[1] 95-97°C); 1H NMR (270 MHz, $CDCl_3$): δ3.80 (s, CH_3, 6H), 4.23 (dd, H-2 and H-4, J = 3.5 Hz, 3.6 Hz, 2H), 4.30-4.35 (m, H-3, 1H), 4.40-4.42 (m, H-1 and H-5, 2H), 4.53 (d, CH_2 J = 11.9 Hz, 2H), 4.58 (d, CH_2, J = 11.9 Hz, 2H), 5.25 (s, =CH_2, 2H), 5.56 (s, O_3CH H), 6.82-6.85 (m, Ar-H, 4H), 7.22-7.25 (m, Ar-H, 4H); 13C NMR (68.8 MHz, $CDCl_3$): δ 55.17 (OCH_3), 68.97 (Ins C), 71.09 (CH_2), 74.29 and 73.58(Ins C), 103.69 (O_3CH), 113.82, 129.47, 137.19 and 159.40 (Ar-C), 114.29 (=CH_2), 129.83 (Ins C=); MS (m/z): $(M+H)^+$, 427 (100%); analysis (calcd., found for $C_{24}H_{26}O_7$): C (67.59, 67.5), H (6.15, 6.2).

(1,3,5/2,4,6)-6-Hydroxymethyl-1,3,5-*O*-methylidyne-2,4-di-*O*-(4-methoxybenzyl)-cyclohexane 1,2,3,4,5-pentol (**25**)

To the alkene **24** (5.00 g, 11.72mmol) was added 9-borabicyclononane (50 mL of a 0.5 M solution in THF, 25 mmol) at room temperature under N_2. The mixture was stirred for 2 h at 50 °C under N_2. The mixture was then cooled to 0°C, then ethanol (17 mL), 6 M aqueous NaOH (4.2 mL) and 30% H_2O_2 were added dropwise. The mixture was then stirred at 50°C for a further 30 min. The mixture was cooled and water (20 mL) was added. The aqueous layer was saturated with K_2CO_3 and the organic layer was removed, dried with $MgSO_4$ then evaporated. Chromatography (EtOAc/hexane 1:1) gave the title compound (3.88 g, 74%) as a white solid: mp: 79-81°C (EtOH) (lit.[1] 81-82°C); 1H NMR (400 MHz, $CDCl_3$): δ 1.72-1.76 (m, CH_2O*H,* 1H), 2.95-2.99 (m, H-6, 1H), 3.79 (s, CH_3, 6H), 4.06-4.08 (m, CH_2OH, 2H), 4.26-4.28 (m, H-1 and H-5, 2H), 4.34-4.36 (m, H-2 and H-4, 2H), 4.48 (d, CH_2, *J* = 10.7 Hz, 1H), 4.49 (d, CH_2, *J* = 11.3 Hz, 1H), 4.51-4.53 (m, H-3, 1H), 4.57 (d, CH_2, *J* = 11.3 Hz, 1H), 4.58 (d, CH_2, *J* = 11.0 Hz, 1H), 5.57 (s, O_3CH, 1H), 6.79-6.83 (m, Ar-H, 4H), 7.15-7.19 (m, Ar-H, 4H).

(1,3,5/2,4,6)-6-hydroxymethyl-2,4-di-*O*-(4-methoxybenzyl)-cyclohexane 1,2,3,4,5-pentol (**26**)
The alcohol **25** (3.88 g, 8.73 mmol) in MeOH (150 mL)/1 M Hydrochloric acid (15 mL) was heated to 50 °C. After 40 min, concentrated aqueous ammonia solution (20 mL) was added and the mixture stirred at room temperature for 1h. Evaporation then chromatography ($CHCl_3$/MeOH 5:1 then 1:1) gave the title compound (2.81 g, 74%) as a white solid: mp 135-136.5 °C (lit[1] 136-137 °C); 1H NMR (270 MHz, $[^2H]_6$-DMSO): δ 1.25 (tt, H-6, *J* = 10.7 Hz, 2 Hz, 1H) 3.09 (dd, H-2 and H-4, *J* = 9.5 Hz, 9.2 Hz, 2H), 3.21 (dt, H-3, *J* = 5.5 Hz, 9.2 Hz, D_2O ex. gives t, *J* = 9.5 Hz, 1H), 3.29-3.33 (m, H-1 and H-5, D_2O ex. gives dd, *J* = 10.4 Hz, 9.5 Hz, 2H), 3.68-3.71 (m, C*H*$_2$OH, 2H), 3.73 (s, OCH_3, 6H), 4.27 (t, CH_2O*H*, *J* = 5.2 Hz, 1H), 4.72 (s, CH_2, 4H), 4.74 (d, OH-1 and OH-5, *J* = 5.8 Hz, 2H), 4.92 (d, OH-3, *J* = 5.5 Hz, 1H), 6.85-6.88 (m, Ar-H, 4H) 7.34-7.36 (m, Ar-H, 4H).

DL-(1,3,5/2,4,6)-1,7-*O*-Benzylidene-6-hydroxymethyl-2,4-di-*O*-(4-methoxybenzyl)-cyclohexane-1,2,3,4,5-pentol (**27**)
To the tetrol **26** (2.78 g, 6.39 mmol) in DMF (15 mL) was added benzaldehyde dimethyl acetal (1.2 g, 7.88 mmol) and PTSA (50 mg). The flask was fitted with a 25 cm air condenser connected to a water pump and the solution was stirred at 70 °C under reduced pressure. After 50 min, the mixture was cooled to room temperature and triethylamine (2 mL) was added. After 1h, the solvents were evaporated and the residue dissolved in CH_2Cl_2 then washed with water (80 mL), brine (80 mL) then dried with $MgSO_4$. Evaporation, then recrystallization (EtOAc/hexane) gave the title compound (2.76 g, 82%) as a white solid: mp: 160-161 °C (lit.[1] 158-160°C); 1H NMR (400 MHz, $CDCl_3$): δ 1.93 (dddd, H-6, *J* =10.7 Hz, 10.7 Hz, 10.7 Hz, 4.5 Hz, 1H), 2.34 (d, OH, *J* = 1.8 Hz, 1H), 2.66 (d, OH, *J* = 1.8 Hz, 1H), 3.23 (ddd, H-3, *J* = 1.8 Hz, 8.9 Hz, 11.0 Hz, D_2O ex. gives dd, *J* = 8.9 Hz, 11.0 Hz, 1H), 3.33 (dd, H-4, *J* = 8.9 Hz, 8.9 Hz, 1H), 3.51-3.71 (m, H-1, H-2, H-3 and CH_2, 4H), 3.78 (br s, OCH_3, 6H,), 4.50 (dd, CH_2, *J* = 4.3 Hz, 11.0 Hz, 1H), 4.60 (d, CH_2, *J* = 11.0 Hz, 1H), 4.63 (d, CH_2, *J* = 11.0 Hz, 1H), 4.96

(d, CH_2, J = 11.3 Hz, 1H), 4.97 (d, CH_2, J = 11.3 Hz, 1H), 5.52 (s, C*H*Ph, 1H,), 6.84-6.90 (m, Ar H, 4H), 7.24-7.30 (m, Ar H, 4H), 7.33-7.52 (m, Ar H, 5H).

DL-(1,3,5/2,4,6)-1,3-Di-*O*-benzyl-5,7-*O*-benzylidene-6-hydroxymethyl-2,4-di-*O*-(4 methoxybenzyl) cyclo-hexane 1,2,3,4,5-pentol (**16**)

To the diol **27** (100 mg, 0.19 mmol) in dry DMF (10 mL) was added sodium hydride (25 mg of a 60% dispersion in mineral oil, 0.625 mmol) at room temperature. After 30 min, benzyl bromide (0.05 mL, 0.46 mmol) was added and the mixture was stirred overnight. Water (5 mL) was added then the solvents were evaporated. The residue was dissolved in CH_2Cl_2 (20 mL) and washed with brine (20 mL) then dried with $MgSO_4$. Evaporation gave a white solid which was washed with pentane then recrystallized (EtOH) to give the title compound (121 mg, 90%) as white crystals: mp: 135-137 °C (lit.[1] 135-137 °C); 1H NMR (270 MHz, $CDCl_3$): δ 1.97-2.00 (m, H-6, 1H), 3.21-3.25 (m, H-1, 1H) 3.43-3.71 (m, H-2, H-3, H-4, H-5, and CH_2 axial, 5H), 3.77 (s, OCH_3, 3H), 3.78 (s, OCH_3, 3H), 4.42 (dd, CH_2 equatorial, J = 11.0 Hz, 4.4 Hz, 1H), 4.50-4.98 (m, Ar-CH_2, 8H), 5.48 (s, CHPh, 1H), 6.77-6.84 (m, Ar-H, 4H), 7.20-7.51 (m, Ar-H, 19H).

Spectroscopic and characterization data for 1-p, 5-s, 5-p and synthetic intermediates

Scyllo-inositol 1,2,3,5-tetrakisphosphate (**1-p**).

2,4-Di-*O*-*p*-methoxybenzyl-1,3,5-*O*-methylidyne *scyllo*-inositol (**13**).

mp: 125-126 °C (from ethyl acetate/hexane or ethanol); 1H NMR (270 MHz, $CDCl_3$): δ 3.79 (s, 6H), 4.10 (d, J = 12.45 Hz, 1H, D_2O ex. 6-OH), 4.34-4.42 (m, 3H), 4.43-4.48 (2H), 4.56 (AB system, 4H), 4.56 (m, buried 1H), 5.49 (s, 1H), 6.77-6.84 (m, 4H), 7.08-7.16 (m, 4H); 13C NMR (68 MHz, $CDCl_3$): δ 55.15 (q), 66.71, 68.73, 69.18, 72.85 (4d), 71.36 (t), 102.40 (d, O_3*C*H), 113.83 (d), 129.56 (d), 129.72 (s), 159.42 (s); MS (m/z) (+ve ion FAB): 431 [$(M+H)^+$, 1%], 309 (8%), 121 [$(CH_2C_6H_4OCH_3)^+$, 100%]; analysis (calc., found for $C_{23}H_{26}O_8$): C (64.18, 63.9) H (6.09, 6.08).

1,3-Di-*O*-*p*-methoxybenzyl-*scyllo*-inositol (**14**).

mp: 161-163 °C (from ethyl acetate/methanol); 1H NMR (270 MHz, d_6-DMSO,): δ 2.98-3.19 (m, 5H), 3.29 (m, D_2O ex. gives t, J = 9.2 Hz, 1H), 3.73 (s, 6H), 4.70 (s, 4H), 4.84 (d, J = 3.5 Hz, D_2O ex. 3H), 4.98 (d, J = 5.3 Hz, 1H), 6.86 (d, J = 8.4 Hz, 4H), 7.34 (d, J = 8.4 Hz, 4H); 13C NMR (68 MHz, d_6-DMSO,): δ 55.09 (q), 73.43 (t), 73.67 (d), 73.79 (d), 74.50 (d), 82.95 (d), 113.33 (d), 129.27 (d), 131.75 (s), 158.49 (s); MS (m/z) (+ve ion FAB): 419 [$(M-H)^+$, 3%], 299 (12), 149 (15), 121 [$(CH_2C_6H_4OCH_3)^+$, 100%]; MS (m/z) (-ve ion FAB): 573 [$(M+NBA)^-$, 95%], 419 [$(M-H)^-$, 100], 291 (43), 118 (32); analysis (calcd., found for $C_{22}H_{28}O_8$): C (62.5, 62.85); H 6.69, 6.71).

4,6-Di-*O*-*p*-methoxybenzyl-*scyllo*-inositol 1,2,3,5-tetrakis[bis(2-cyanoethyl)phosphate] (**15**).
1H NMR (400MHz, $CDCl_3$): δ 2.36-2.58 (m, 8H), 2.72 (br t, *J* ~ 6 Hz, 4H), 2.84 (br t, *J* ~ 6 Hz, 4H), 3.80 (s, 6H), 3.86 (dd, *J* = 7.6 Hz, 7.6 Hz, 2H), 3.97-4.47 (m, 16H), 4.61-4.71 (m, 4H), 4.79 (AB system, J_{AB} = 11.3 Hz, 4H), 6.90 (d, *J* = 8.5 Hz, 4H), 7.39 (d, *J* = 8.5 Hz, 4H); 13C NMR (100 MHz, $CDCl_3$,): δ 19.32 (t), 19.55 (t), 19.70 (t), 55.34 (q), 62.67 (t), 62.85 (t), 63.08 (t), 63.28 (t), 73.92 (t), 76.50, 77.27, (2d), 78.20 (d), 78.60 (d), 113.81 (d), 116.66, 116.90, 117.12, 117.43 (4s), 128.93 (d), 129.35 (s), 159.34 (s); 31P NMR (162 MHz, $CDCl_3$): δ –2.79 (s, 1P), –3.11(s, 3P); MS (m/z) (+ve ion FAB): 1165 [$(M+H)^+$, 40%], 1043(60), 281(80), 121 [$(CH_2C_6H_4OCH_3)^+$, 100%]; MS (m/z) (-ve ion FAB): 1367(30), 1330(80), 1110(100), 203[$((NCC_2H_5O)_2PO_2)^-$, 45%].

Scyllo-inositol 1,2,3,5-tetrakisphosphate (**1-p**).
1H NMR (400 MHz, D_2O, pH 4): δ 3.48 (dd, *J* = 9.76 Hz, 8.55 Hz, 2H), 3.83 (dt, *J* = 8.85 Hz, 9.16 Hz, 1H), 3.90-4.02 (m, 3H); 31P NMR (162 MHz, D_2O pH 4): δ 0.11 (d, J_{HP} = 8.85 Hz, 2P), 0.38 (d, J_{HP} = 8.85 Hz, 1P), 0.53 (d, J_{HP} = 8.85 Hz, 1P); MS (m/z) (+ve ion FAB): 102 [$(C_2H_5)_3NH^+$, 100%]; MS (m/z) (-ve ion FAB): 999 [$(2M+H)^-$, 80%], 499 [M^-, 100%]; HRMS (m/z) FAB^-: $[M]^-$ calcd. for $C_6H_{15}O_{18}P_4^-$ 498.921; found, 498.919.

DL-6-deoxy-6-hydroxymethyl-*scyllo*-inositol 1,2,4-trisphosphate (racemic **5-s**).

DL-6-Deoxy-1,3-di-*O*-benzyl-6-benzyloxymethyl-2,4-di-*O*-*p*-methoxybenzyl-*scyllo*-inositol (**17**).
1H NMR (270 MHz, $CDCl_3$): δ 1.67 (m, 1 H), 2.66 (d, *J* = 2.0 Hz, D_2O ex., 1H), 3.37 (dd, *J* = 9.2 Hz, 9.2 Hz, 1H), 3.49-3.85 (m, 6H), 4.41-4.96 (m, AB systems, 10H), 6.78-6.88 (m, 4 H) 7.18-7.36 (m, 19H); 13C NMR (68 MHz, $CDCl_3$): δ 45.46 (d), 55.16 (q,), 65.65 (t,), 69.48 (d), 73.06, 75.09, 75.35, 75.59 (4t), 77.44, 83.07, 85.22, 85.92 (4d), 113.73, 113.93 (2d), 127.40, 127.45, 127.53, 127.66, 127.78, 128.85, 128.33 (7d), 130.74 (s), 138.27, 138.50, 138.56 (3s), 159.08, 159.26 (2s); MS (m/z) (+ve ion FAB, rel intensity): 705[$(M+H)^+$, 24%], 584[$(M-PMB)^+$, 28%], 211(80), 121[$(CH_2C_6H_4OMe)^+$, 100%]; MS (m/z) (-ve ion FAB, rel intensity): 857 [$(M+NBA)^-$, 100%], 703 [$(M-H)^-$, 40%], 470(78), 303(60), 140(80), 121(80); analysis (calcd., found for $C_{44}H_{48}O_8$): C (74.98, 74.7); H (6.80, 6.86).

DL-6-Deoxy-1,3-di-*O*-benzyl-6-benzyloxymethyl-*scyllo*-inositol (**18**).
R_f 0.24 (ethyl acetate/hexane 1:1); mp: 93-95 °C (from hexane); 1H NMR (400 MHz $CDCl_3$): δ 1.70 (dddd, *J* = 10.7 Hz, 10.7 Hz, 4.4 Hz, 2.4 Hz, 1H), 2.59 (d, *J* = 1.95 Hz, D_2O ex.1 H), 2.83 (br s, D_2O ex. 1H), 3.19 (d, *J* = 2.9 Hz, D_2O ex. 1H), 3.22 (dd, *J* = 9.76 Hz, 9.28 Hz, 1H), 3.38-3.48 (m, 2H), 3.57-3.68 (m, 3H), 3.85 (dd, *J* = 9.28 Hz, 2.4 Hz, 1H), 4.46, 4.50 (AB q, J_{AB} = 11.7 Hz, 2H), 4.58, 4.78 AB q, J_{AB} = 10.7 Hz, 2H), 4.86 (s, 2H), 7.24-7.38 (m,15H); 13C

NMR (68 MHz, $CDCl_3$): δ 44.92 (d), 66.60 (t), 70.58 (d), 73.25, 74.79, 74.83 (3t), 76.69, 77.05, 77.65, 81.88 (4d), 127.68, 127.75, 127.86, 127.92, 127.99, 128.39, 128.43, 128.54 (9d), 137.95, 138.40, 138.53 (3s); MS (m/z) (+ve ion FAB, rel intensity): 465 [$(M+H)^+$, 6%], 181(15), 91 [$(C_7H_7)^+$, 100%]; MS (m/z) (-ve ion FAB, rel intensity): 617 [$(M+NBA)^-$, 80%], 463 [$(M-H)^-$, 100%]; analysis (calcd., found for $C_{28}H_{32}O_6$): C (72.39,72.3); H 6.94, 6.90).

DL-6-Deoxy-3,5-di-*O*-benzyl-6-benzyloxymethyl-*scyllo*-inositol1,2,4-tris(dibenzylphosphate) (**19**).
mp: 87.5 - 88.5 °C (from hexane); R_f 0.20 (chloroform/acetone 10:1); 1H NMR (400 MHz, $CDCl_3$): δ 1.93 (br t, J = 10.7 Hz, 1H), 3.63 (1H, dd, J = 8.9 Hz, 8.6 Hz), 3.71-3.77 (2H, m), 3.84 (1H, dd, J = 9.5 Hz, 2.1 Hz), 4.18, 4.44 (AB q, J_{AB} = 11.6 Hz, 2H), 4.42 (half of AB system, 1H), 4.53-5.07 (AB systems of $OCH_2C_6H_5$, C-1-H, C-2-H, C-4-H, 18H), 6.93-7.01 (m, C_6H_5, 6H), 7.08-7.26 (m, 37H), 7.39-7.41 (m, 2H); 13C NMR (100 MHz, $CDCl_3$): δ 44.75 (d), 63.66 (t), 69.24, 69.29, 69.35, 69.40, 69.71, 69.77 (6t), 72.56, 73.90, 74.34 (3t), 74.98, 75.20, 78.86, 80.23, 81.84 (5d), 127.05, 127.41, 127.52, 127.61, 127.80, 127.91, 127.98, 128.02, 128.11, 128.18, 128.27, 128.33, 128.38, 128.47 (14d), 135.77, 135.83, 135.90, 135.97, 136.03, 136.10 (6s), 138.06, 138.20, 138.24 (3s); 31P NMR (162 MHz, $CDCl_3$, 1H-decoupled): δ -2.12 (s, 1P), -1.67(s, 1P), -1.53 (s, 1P); MS (m/z) (+ve ion FAB, rel intensity): 1245 [$(M+H)^+$, 65%], 271(10), 181(10), 91 [$(C_7H_7)^+$, 100%]; MS (m/z) (-ve ion FAB, rel intensity): 1397 [$(M+NBA)^-$, 80%], 1153 [$(M-C_7H_7)^-$, 100%], 277 [$(C_6H_5CH_2O)_2P(O)O^-$, 100%]; analysis (calcd., found for $C_{70}H_{71}O_{15}P_3$): C (67.52, 67.4), H (5.75, 5.64).

DL-6-Deoxy-6-hydroxymethyl-*scyllo*-inositol 1,2,4-trisphosphate (racemic **5-s**).
1H NMR (400 MHz, D_2O): δ 1.47 (dddd, J = 11 Hz, 11 Hz, 2 Hz, 1H), 3.38 (dd, J = 9.5 Hz, 9.2 Hz, 1H), 3.48 (dd, J = 11 Hz, 1H), 3.68 (ABX, 2H), 3.80 (ddd, J = 9.5 Hz, 9.2 Hz, 8.5 Hz, 1H), 3.87 (ddd, J = 10.4 Hz, 10.4 Hz, 10.4 Hz, 1H), 4.00 (ddd, J = 9.2 Hz, 9.2 Hz, 9.2 Hz, 1H). 31P NMR (162 MHz, D_2O): δ 0.15 (d, J_{HP} = 10.0 Hz, 1P), 0.45 (d, J_{HP} = 8.1 Hz, 1P), 0.96 (d, J_{HP} = 9.0 Hz, 1P,); MS (m/z) (+ve ion FAB, rel intensity): 102 [$(C_2H_5)_3NH^+$, 100%]; MS (m/z) (-ve ion FAB, rel intensity): 867 [$(2M-H)^-$, 10%], 433 [$(M-H)^-$, 100%], 159(10), 97 [$H_2PO_4^-$, 35%]; HRMS (m/z) (FAB^-): $(M-H)^-$ calcd. for $C_7H_{16}O_{15}P_3^-$ 432.9702; found 432.9708.

DL-6-Deoxy-6-phosphoryloxymethyl-*scyllo*-inositol 1,2,4-trisphosphate (racemic **5-p**).

DL-6-Deoxy-6-hydroxymethyl-1,3-di-*O*-benzyl-*scyllo*-inositol (**20**).
mp: 124-125 °C (from EtOAc/hexane); 1H NMR (400 MHz, $[^2H]_6$-DMSO): δ 1.26 (t, H-6 J = 10.7 Hz, 1H), 3.02 (t, J = 9.3 Hz, 1H), 3.26-3.37 [m, 3H, D_2O ex. gives 3.21 t, J = 9.2 Hz,1H), 3.32 (t, J = 10.4 Hz, 1H), 3.35 (1H, t, H-1 J 10.1 Hz)], 3.44-3.46 (1H, m, H-2 D_2O ex gives t, J 9.2 Hz), 3.67-3.76 (m, 2H), 4.36 (t, J = 4.4 Hz, 1H), 4.59 (d, J = 11.2 Hz, 1H), 4.66 (d, J = 4.9 Hz, 1H), 4.79 (d, J = 11.7 Hz, 1H), 4.83 (d, J = 11.7 Hz, 1H), 4.88 (d, J = 4.9 Hz, 1H), 4.91

(d, J = 11.2 Hz, 1H), 5.10 (d, J = 5.8 Hz, 1H), 7.23-7.46 (m, 10H); 13C NMR (100 MHz, $[^2H]_6$-DMSO,): δ 46.60 56.29, 68.97, 76.67, 76.77, 77.80, 83.19, 73.74, 127.06, 127.19, 127.69, 127.95, 128.17, 139.65, 139.84; analysis (calcd., found for $C_{21}H_{26}O_6$): C (67.36, 67.1), H, (7.00,7.10).

DL-6-deoxy-6-(dibenzyloxyphosphoryloxy)methyl-3,5-di-*O*-benzyl-*scyllo*-inositol 1,2,4-tris(dibenzylphosphate) (**21**).
1H NMR (400 MHz; $CDCl_3$) δ 2.02-2.06 (m, 1H) 3.51-3.64 (m, 2H), 4.47-4.50 (m, 2H), 4.59-5.07 (m, 23H), 6.97-7.40 (m, 50H); 31P NMR (162 MHz; $CDCl_3$) δ −0.81 (s, 1P), −1.58 (s, 1P), −1.85 (s, 1P), −2.18 (s, 1P); HRMS (m/z) (FAB+): $(M+H)^+$ calcd. for $C_{77}H_{79}O_{18}P_4$, 1415.4217; found 1415.4204; analysis (calcd., found for $C_{77}H_{78}O_{18}P_4$): C (65.34, 64.8), H (5.55, 5.6).

DL-6-Deoxy-6-phosphoryloxymethyl-*scyllo*-inositol 1,2,4-trisphosphate (racemic **5-p**).
^{1}H NMR (400 MHz, D_2O): δ 1.61 (t, J = 10.7 Hz, 1H), 3.39 (t, J = 9.2 Hz, 1H), 3.52 (t, J = 10.1 Hz, 1H), 3.81 (q, J = 8.9 Hz, 1H), 3.92-3.99 (m, 3H), 4.05-4.12 (q, J = 10.1 Hz, 1H); 31P NMR (D_2O, 162 MHz): δ 0.64 (s, 1P), 0.39 (s, 1P), 0.47(s, 1P), 0.62 (s, 1P); HRMS (m/z) (FAB^-): $(M-H)^-$ calcd. for $C_7H_{17}O_{18}P_4$, 512.9365; found 512.9367.

Reaction of 2-FITC-InsP$_3$ with IP3K

150 nM IP3K-KD was incubated with 10 μM 2-FITC-InsP$_3$ and 1 mM ATP under ATP-regenerating conditions in 20 mM HEPES pH 7.5, 1mM $MgCl_2$ or in 20 mM HEPES pH 7.5, 1 mM EDTA for 2 hours at 30 °C.

HPLC of 2-FITC-InsP$_3$ and reaction products

2-FITC-InsP$_3$ and analogs were resolved by HPLC on a 2mm x 250 mM Dionex AS11 column, with guard column 2 x 50 mm, eluted with a gradient of NaOH at a flow rate of 0.4 mL/min. The gradient was delivered from reservoirs containing water, A, and 225 mM NaOH, B, according to the schedule: time (min), % B; 0, 0; 20, 100; 30, 100; 31, 0, 0. Analogs were detected with a Jasco FP-920 fluorescence detector with settings: excitation, 485 nm; emission, 515 nm; gain x10 (Supplementary Fig. 5a). Additionally, 2-FAM-InsP$_3$ and 2-FAM-InsP$_5$ were synthesized as described[2] and were analyzed by HPLC without enzyme treatment (Supplementary Fig. 5b).

Supplementary Figures

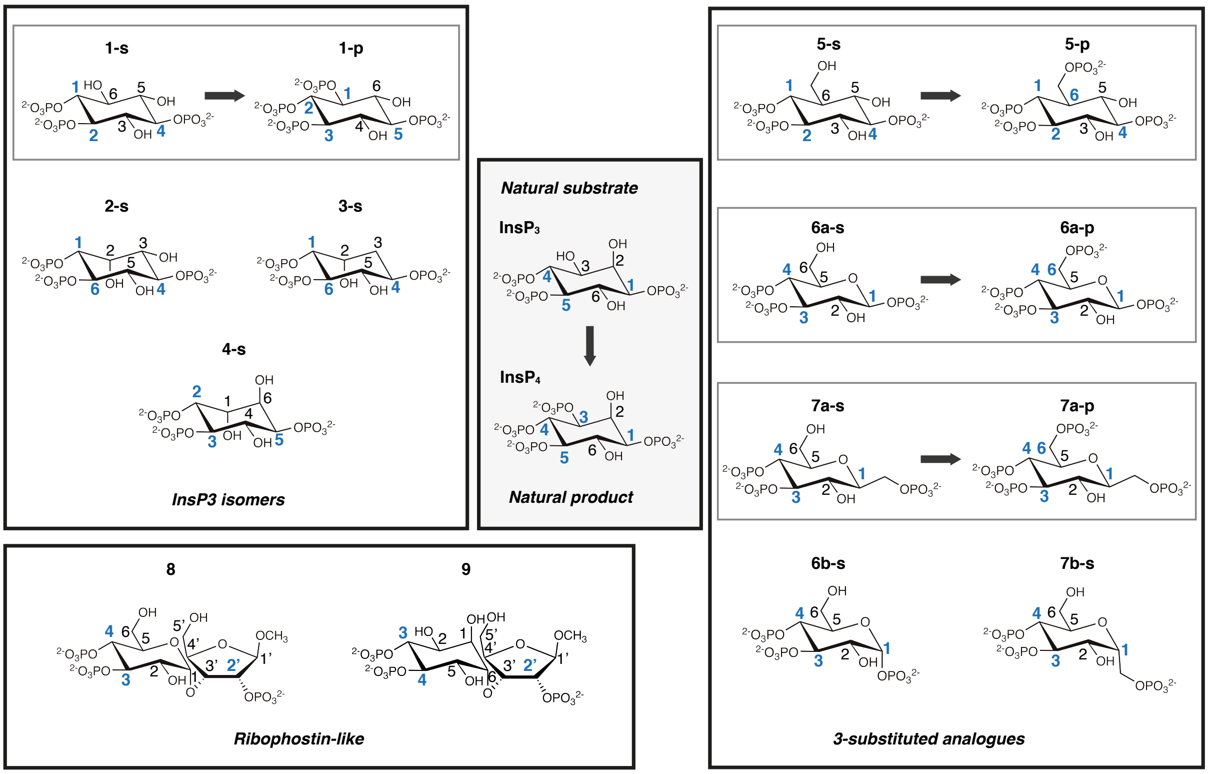

Supplementary Figure 1. InsP analogues used in this work showing their systematic numbering. Same compounds as shown in Fig. 1, but the numbering here corresponds to each compound instead of being referred to $InsP_3$ positions. Numbers in blue show the phosphates that mimic $InsP_3$/$InsP_4$ phosphates as shown in the experimental structures in all cases except compounds **7b-s**, **8** and **9**.

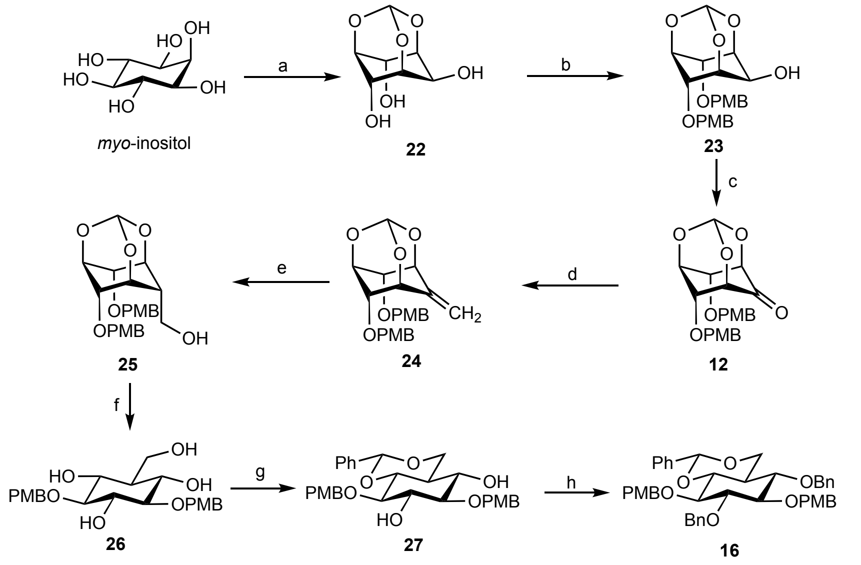

Supplementary Figure 2. Synthesis of DL-(1,3,5/2,4,6)-1,3-Di-*O*-benzyl-5,7-*O*-benzylidene-6-hydroxymethyl-2,4-di-*O*-(4-methoxybenzyl)cyclohexane 1,2,3,4,5-pentol (16). Reagents and conditions: a) $(EtO)_3CH$, DMF, *p*-TSOH, 140 °C; b) NaH, PMBCl, DMF, r.t.; c) DMSO, $(COCl)_2$, CH_2Cl_2, Et_3N, –60 °C; d) CH_3PPh_3Br, *t*-BuOK, THF, reflux: e) i) 9BBN-H, THF, 50 °C; ii) OH^-, H_2O_2; f) i. 1M HCl/MeOH 1:10, reflux; ii. conc. aqueous NH_3; g) $C_6H_5CH(OMe)_2$, DMF, *p*-TSOH; h) NaH, BnBr, DMF. Bn = benzyl, PMB = *p*-methoxybenzyl. All asymmetrical compounds are racemic.

a

		Distances		
		1 - 4 (Å)	2 - 5 (Å)	3 - 6 (Å)
Substrate mimic	**$InsP_3$**	**8.0**	6.0	5.7
	**1-s**	8.3	**6.9**	**5.6**
	**6a-s**	8.2	**5.6**	6.4
	**7a-s**	9.2	**5.6**	6.4
		1 - 4 (Å)	2 - 5 (Å)	3 - 6 (Å)
Product mimic	**$InsP_4$**	8.3	6.1	6.7
	**1-p**	8.3	**6.9**	6.8
	**6a-p**	8.4	**5.7**	7.2
	**7a-p**	**9.4**	5.6	**7.2**

b **c**

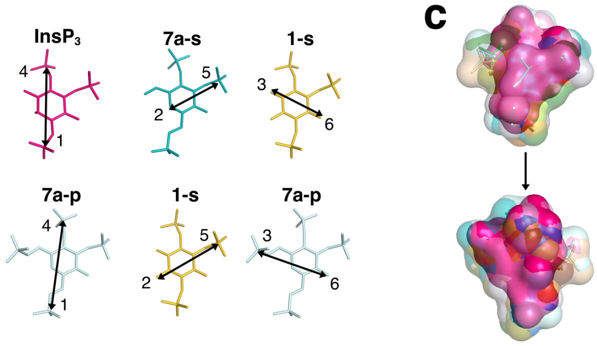

d

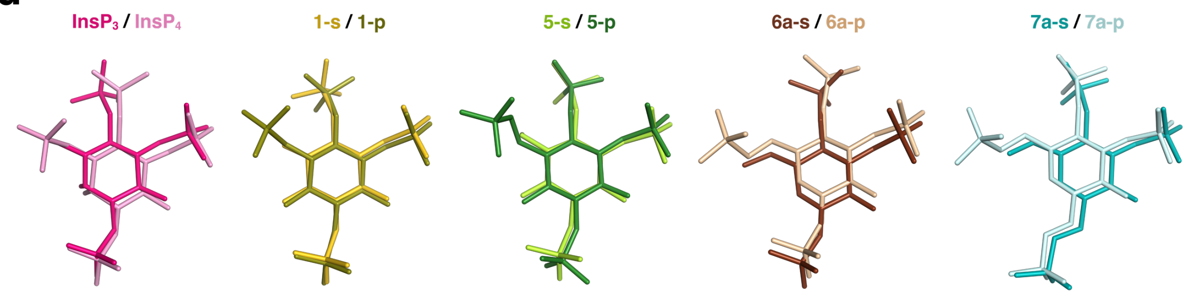

Supplementary Figure 3. Comparison of positioning of $InsP_3$ and $InsP_4$ mimics in the IP3KA active site. a, Table with selected ligands to show minimum and maximum distances (highlighted in bold letters) between the substituents of InsP analogues laying in opposite positions (1-4; 2-5 and 3-6). For the calculations we have considered the center of the substituent as the phosphorus atom when the substituent is a phosphate, oxygen atom when it is a hydroxyl or the oxygen of the ring in a glucose-based analogue. **b,** Representation of ligands that exhibit the shortest (up) and the longest (down) distances expressed in panel (a). **c,** Surface representation of InsP analogues (transparent) superimposed on $InsP_3$ (pink) as found in IP3K complexes, showing the available room for protein-ligand recognition. **d,** Substrate/product pairs representation to show their fitting in a very similar site.

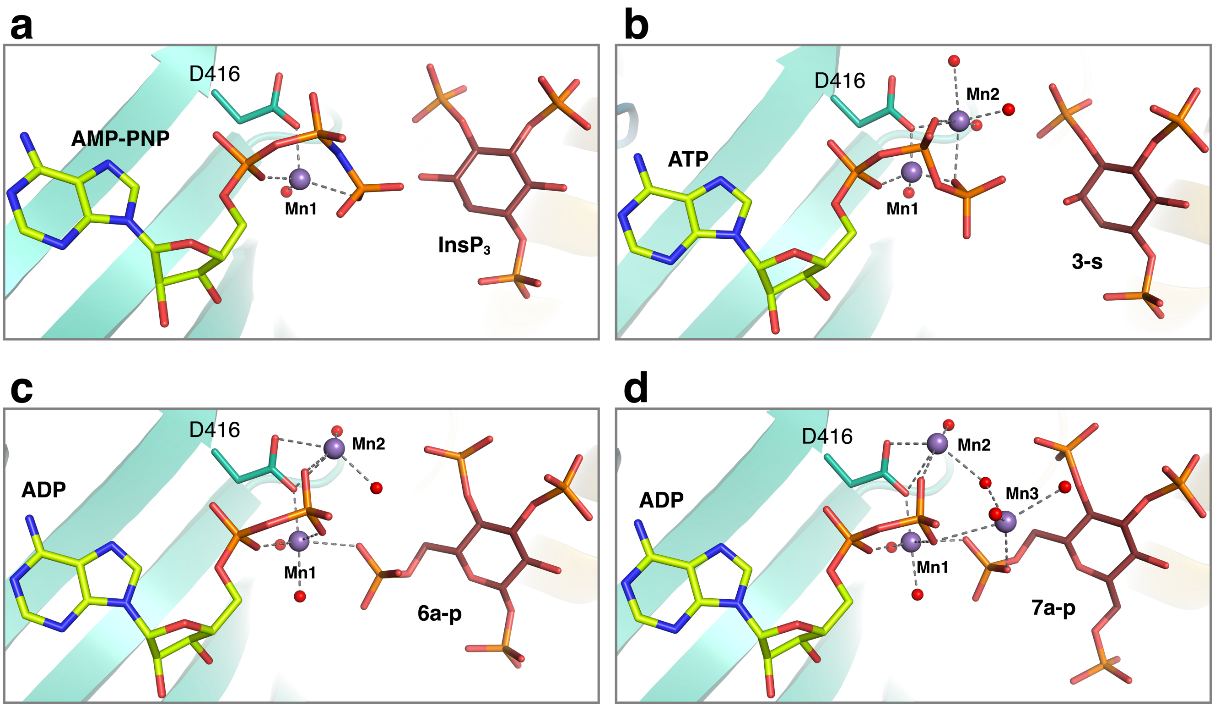

Supplementary Figure 4. IP3K uses up to three Mg^{2+} positions for ligand binding and catalysis. IP3K is able to bind up to three metal positions in the active site. The color code is similar to Figure 3. **a**, The IP3K/$InsP_3$ complex shows one metal position (Mn1) clearly associated with ATP binding and in charge of orienting the Pγ. Mn1 is kept in almost all complexes with the exception of compounds **6a-s** (soaked with Mg^{2+} instead of Mn^{2+}) and **4s**. **b,** Compounds exhibiting an axial hydroxyl in the pseudo 3-position, such as compound **3-s**, present a second metal position (Mn2); however, such analogues are not substrates of the reaction and this metal is probably just stabilizing both ATP and InsP analogue binding to IP3K. **c, d,** Interestingly, complexes of IP3K with products formed *in situ* in the crystallization experiment also exhibit two (Mn1 + Mn2) (c, **6a-p**) or three (Mn1 + Mn2 + Mn3) (d, **7a-p**) metal positions. Note that the Mn2 position displays a shift and different water coordination sphere when we look at product analogues.

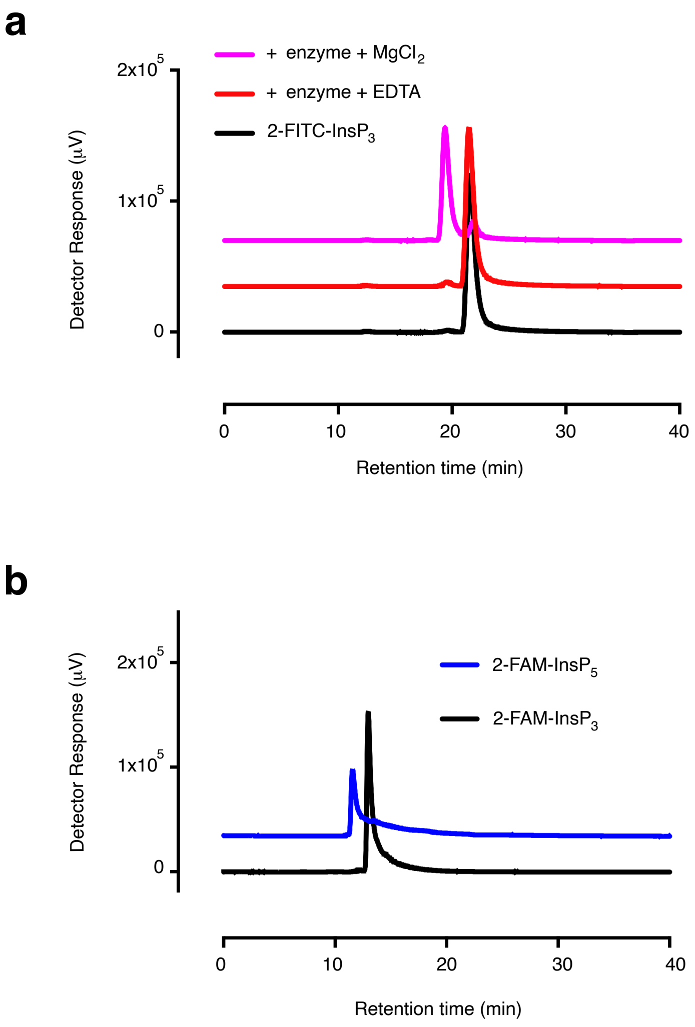

Supplementary Figure 5. **HPLC of the reaction products of IP3K and 2-FITC-InsP$_3$**. **a**, Elution of unreacted FITC-InsP$_3$ (black), or of 2-FITC-InsP$_3$ incubated with IP3K-KD in the presence of $MgCl_2$ (magenta) or presence of EDTA (red). **b**, Elution of unreacted 2-FAM-InsP$_3$ (black) and unreacted 2-FAM-InsP$_5$ (blue). Consistent with b, and[2], in which dephosphorylation of 2-FAM-InsP$_5$ generated a later eluting product, the $MgCl_2$-dependent product of IP3K action on 2-FITC-InsP$_3$ (a) elutes earlier than the substrate itself.

a

All poses

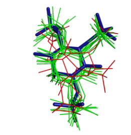

InsP$_3$

Solution	Score	Score / MW	Fit
4	91.28	0.220	green
1	90.24	0.218	green
8	87.00	0.210	green
5	84.66	0.204	green
10	84.08	0.203	green
7	83.37	0.201	green
9	83.32	0.201	green
3	78.39	0.189	red
6	77.08	0.186	green
2	74.77	0.181	red

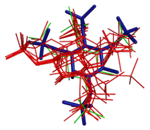

InsP$_4$

Solution	Score	Score / MW	Fit
5	115.62	0.235	green
7	115.17	0.234	red
4	115.16	0.234	red
6	114.25	0.232	red
1	112.82	0.229	red
8	105.53	0.214	red
10	104.51	0.212	red
9	104.27	0.212	red
3	97.33	0.198	red
2	94.87	0.193	red

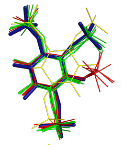

1-s

Solution	Score	Score / MW	Fit
2	119.16	0.288	green
10	118.75	0.287	green
8	114.81	0.277	red
3	114.34	0.276	green
5	114.26	0.276	green
6	113.37	0.274	green
9	112.44	0.272	red
1	108.13	0.261	red
4	104.47	0.252	yellow
7	101.30	0.245	red

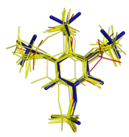

1-p

Solution	Score	Score / MW	Fit
2	127.64	0.259	yellow
6	126.49	0.257	yellow
4	125.05	0.254	yellow
8	124.13	0.252	yellow
5	123.53	0.251	yellow
7	123.30	0.251	yellow
9	122.21	0.248	yellow
3	115.66	0.235	yellow
10	113.42	0.231	red
1	112.63	0.229	yellow

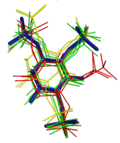

2-s

Solution	Score	Score / MW	Fit
10	129.89	0.314	green
4	128.86	0.311	green
6	127.10	0.307	green
8	127.03	0.307	green
9	122.50	0.296	green
2	120.11	0.290	yellow
3	113.72	0.275	red
7	113.46	0.274	yellow
5	112.25	0.271	red
1	110.64	0.267	red

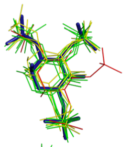

3-s

Solution	Score	Score / MW	Fit
5	128.71	0.323	green
7	125.36	0.315	green
2	119.33	0.300	green
4	117.05	0.294	green
8	114.75	0.288	yellow
3	111.90	0.281	yellow
1	111.88	0.281	green
9	111.45	0.280	red
10	107.14	0.269	green
6	104.22	0.262	yellow

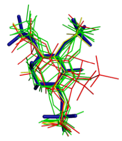

4-s

Solution	Score	Score / MW	Fit
9	118.90	0.287	green
4	114.41	0.276	green
5	114.05	0.275	green
6	112.99	0.273	green
1	110.05	0.266	red
3	109.70	0.265	yellow
10	109.13	0.264	green
7	108.54	0.262	green
2	102.04	0.246	red
8	101.75	0.246	red

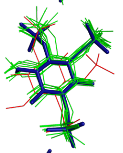

5-s

Solution	Score	Score / MW	Fit
5	131.79	0.308	green
8	128.58	0.300	green
1	128.53	0.300	green
4	123.36	0.288	green
3	122.54	0.286	green
9	121.27	0.283	green
7	120.95	0.283	green
6	120.61	0.282	green
2	113.94	0.266	green
10	96.22	0.225	red

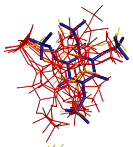

5-p

Solution	Score	Score / MW	Fit
2	111.93	0.221	red
1	104.47	0.206	red
3	104.37	0.206	yellow
4	103.31	0.204	red
5	103.22	0.204	red
9	102.07	0.202	red
6	98.26	0.194	red
10	97.17	0.192	red
7	92.34	0.182	red
8	90.24	0.178	red

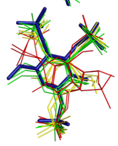

6a-s

Solution	Score	Score / MW	Fit
5	107.01	0.258	green
4	106.18	0.256	green
2	105.53	0.255	red
9	103.62	0.250	red
3	102.38	0.247	yellow
7	102.16	0.247	yellow
6	100.95	0.244	green
10	97.55	0.236	yellow
1	91.27	0.220	green
8	87.07	0.210	red

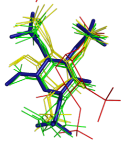

6b-s

Solution	Score	Score / MW	Fit
1	81.80	0.198	yellow
5	81.10	0.196	green
4	80.22	0.194	green
2	78.82	0.190	yellow
3	78.79	0.190	yellow
10	78.12	0.189	green
8	78.08	0.189	yellow
7	75.28	0.182	green
9	75.24	0.182	red
6	74.13	0.179	green

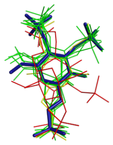

7a-s

Solution	Score	Score / MW	Fit
1	115.73	0.270	green
10	114.71	0.268	green
6	108.66	0.254	green
5	108.38	0.253	green
8	103.48	0.242	green
7	102.96	0.241	green
2	97.84	0.229	yellow
9	96.71	0.226	green
4	95.77	0.224	red
3	86.36	0.202	red

Best fit — Worst fit

b

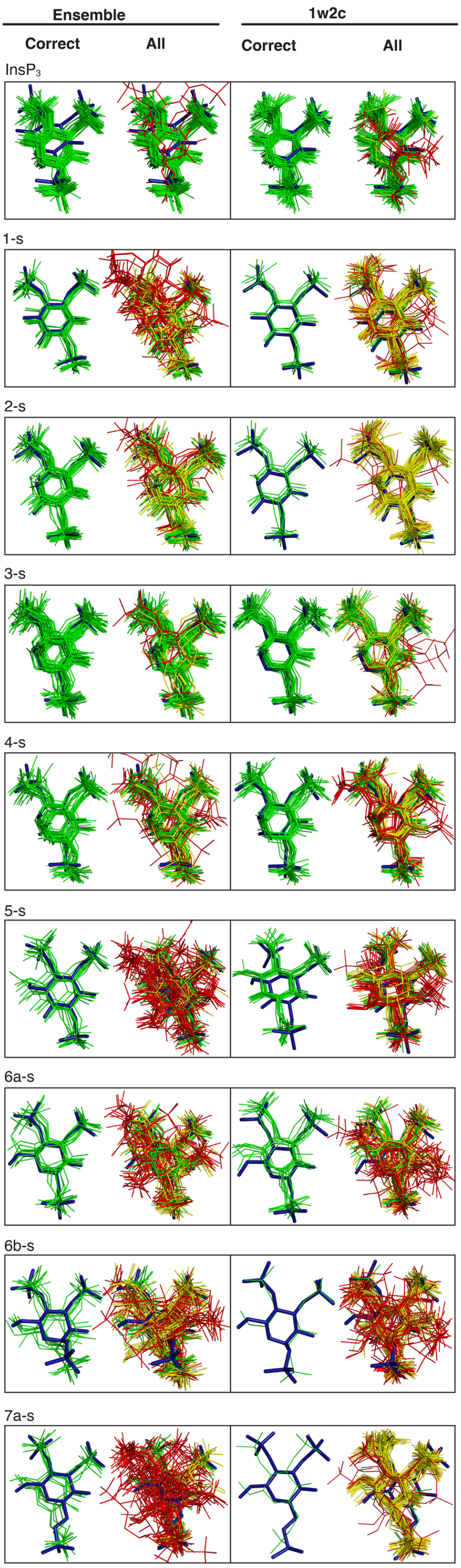

c

Initial Validation	InsP$_3$		1-s		2-s		3-s		4-s		5-s		6a-s		6b-s		7a-s		InsP$_4$		1-p		5-p	
	Ligand (substrates)																		Ligand (products)					
**Protein conformations**																								
IP3K-KD : InsP$_3$	8	0	-		-		-		-		-		-		-		-		-		-		-	
IP3K-KD : 1-s	-		5	1	-		-		-		-		-		-		-		-		-		-	
IP3K-KD : 2-s	-		-		5	2	-		-		-		-		-		-		-		-		-	
IP3K-KD : 3-s	-		-		-		6	3	-		-		-		-		-		-		-		-	
IP3K-KD : 4-s	-		-		-		-		6	1	-		-		-		-		-		-		-	
IP3K-KD : 5-s	-		-		-		-		-		9	0	-		-		-		-		-		-	
IP3K-KD : 6a-s	-		-		-		-		-		-		4	3	-		-		-		-		-	
IP3K-KD : 6b-s	-		-		-		-		-		-		-		5	4	-		-		-		-	
IP3K-KD : 7a-s	-		-		-		-		-		-		-		-		7	1	-		-		-	
IP3K-KD : InsP$_4$	-		-		-		-		-		-		-		-		-		1	0	-		-	
IP3K-KD : 1-p	-		-		-		-		-		-		-		-		-		-		0	9	-	
IP3K-KD : 5-p	-		-		-		-		-		-		-		-		-		-		-		0	1
Correct poses (%)	**80**		**50**		**50**		**60**		**60**		**90**		**40**		**50**		**70**		**10**		**0**		**0**	

Total poses per ligand	10

Ensemble	InsP$_3$		1-s		2-s		3-s		4-s		5-s		6a-s		6b-s		7a-s		InsP$_4$		1-p		5-p	
	Ligand (substrates)																		Ligand (products)					
**Protein conformations**																								
IP3K-KD : InsP$_3$	0	0	0	0	0	0	0	0	0	0	0	0	0	0	0	0	0	0	0	0	0	0	0	0
IP3K-KD : 1-s	2	0	0	0	3	2	0	0	1	1	0	0	0	0	0	1	1	0	0	0	0	0	0	0
IP3K-KD : 2-s	9	0	5	2	10	4	5	4	4	1	1	2	0	3	0	4	0	1	0	0	0	0	0	0
IP3K-KD : 3-s	10	0	0	4	0	4	15	5	14	6	0	1	2	6	2	8	0	0	0	0	0	0	0	0
IP3K-KD : 4-s	0	0	0	3	1	1	3	0	1	2	0	1	0	3	1	4	0	0	0	0	0	0	0	0
IP3K-KD : 5-s	15	0	2	3	7	3	8	0	1	1	6	2	5	2	6	2	4	3	0	0	0	0	0	0
IP3K-KD : 6a-s	1	0	1	0	0	0	1	0	1	0	0	0	1	0	0	1	0	0	0	0	0	0	0	1
IP3K-KD : 6b-s	0	0	0	0	0	0	0	0	0	0	0	0	0	0	0	0	0	0	0	0	0	0	0	0
IP3K-KD : 7a-s	0	0	0	1	0	0	1	0	0	1	3	0	0	4	0	0	1	0	0	0	0	0	0	0
IP3K-KD : InsP$_4$	1	0	0	0	0	0	1	0	0	0	0	0	0	0	0	0	0	0	1	0	1	3	0	5
IP3K-KD : 1-p	8	0	5	1	5	2	4	0	2	1	0	0	1	1	0	1	0	2	5	0	0	5	0	8
IP3K-KD : 5-p	0	0	0	0	0	0	0	0	0	0	0	0	0	0	0	0	0	0	0	0	0	0	0	0
Total poses	46	0	13	14	26	16	38	9	24	13	10	6	9	19	9	21	6	6	6	0	1	8	0	14
Total correct poses	46		13		26		38		24		10		9		9		6		6		1		0	
Correct poses (%)	**92**		**26**		**52**		**76**		**48**		**20**		**18**		**18**		**12**		**12**		**2**		**0**	
Revised correct poses *	46		13		16		23		23		4		8		9		5		5		1		0	
Revised correct poses (%)	**92**		**26**		**32**		**46**		**46**		**8**		**16**		**18**		**10**		**10**		**2**		**0**	

Total poses per ligand	50

*excluding poses of the ligand in which it selects its experimental protein structure

1W2C	InsP$_3$		1-s		2-s		3-s		4-s		5-s		6a-s		6b-s		7a-s	
	Ligand (substrates)																	
IP3K-KD : InsP$_3$	41	1	5	32	5	42	32	12	20	14	12	15	9	15	1	21	1	40
Correct poses (%)	**82**		**10**		**10**		**64**		**40**		**24**		**18**		**2**		**2**	

Total poses per ligand	50

Supplementary Figure 6. Docking validation of IP3K-ligands. a, GOLD best 10 docking solutions (green/yellow/red lines) obtained in the initial validation, i.e. each ligand using its corresponding experimental IP3K-KD structure. The IP3K ligands are shown as compared to the $InsP_3$ position (dark blue sticks). The color code for ligands indicates the quality of the fit in comparison with the experimental structure (green: good overall fit, yellow: phosphates in good place but InsP orientation or torsions are not good, red: not in place). The picture reveals that this method is good for overall prediction in the case of substrate-mimics but not for product mimics. **b**, Superposition of GOLD poses for substrate and substrate mimics, obtained from an ensemble docking and a docking against IP3K-$InsP_3$ protein structure (1w2c). For clarity, two superpositions for each procedure is shown: one showing only the correct poses in green, the other showing all poses obtained (green, red, yellow). The picture reveals a higher trend of the ensemble docking to yield just one cluster coincident with the correct solutions while docking against 1w2c usually present extra clusters of poses in non-correct positions or conformations. Similarly, the deviation from the $InsP_3$-site is predicted in more cases in the ensemble docking. **c,** Number and percentage of GOLD ligand poses (green and yellow) obtained in all the procedures performed: first, obtained in the initial validation (a), second, obtained in the ensemble docking (b left), and third, obtained using 1w2c as protein target (b right). The numbers reveal that overall the ensemble docking is the best procedure to predict unknown binding of an $InsP_3$ mimic to IP3K. This is reflected in the observation that ensemble docking predominantly yields a single cluster in the correct position, while non-correct solutions are dispersed without repetition.

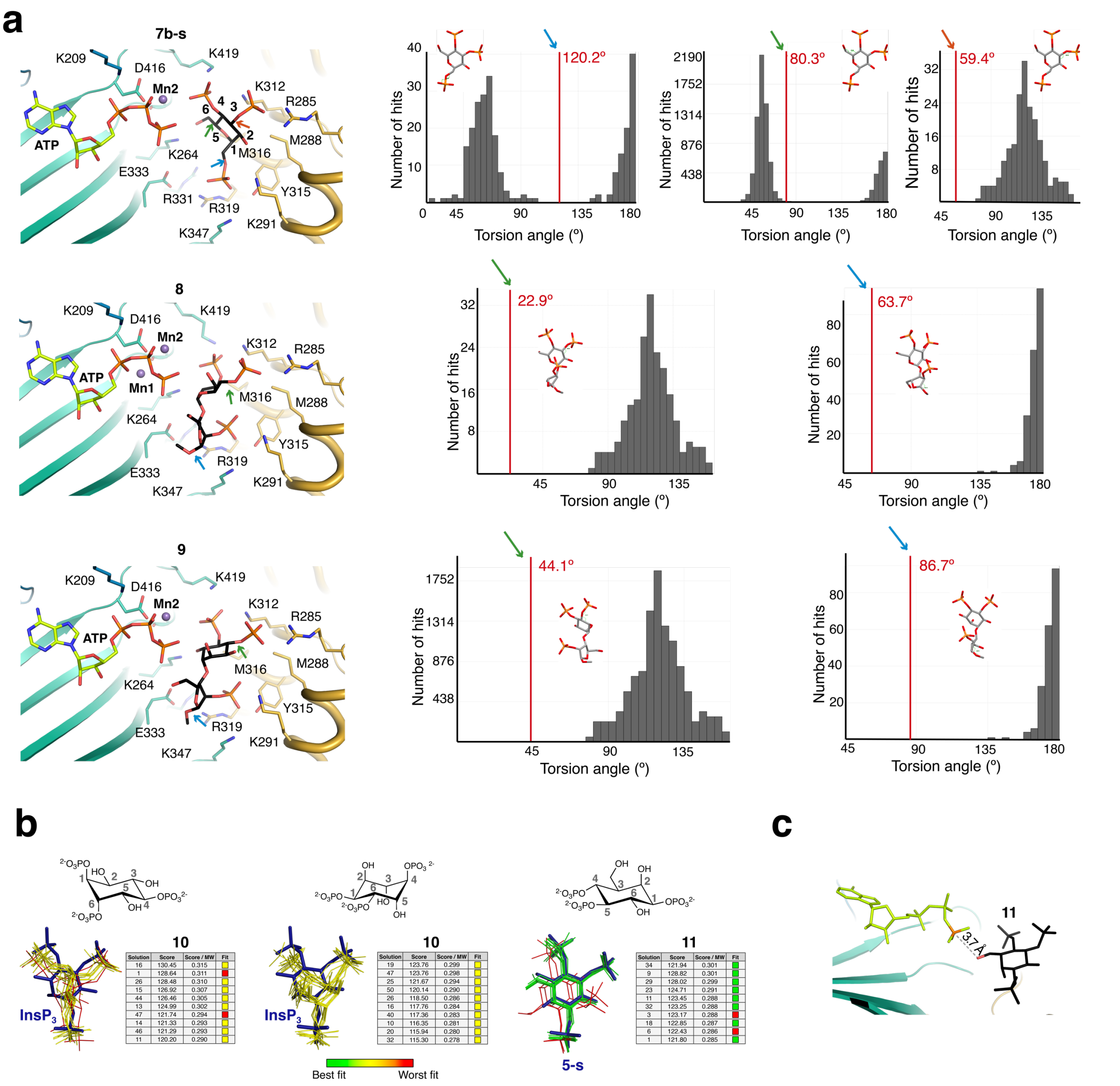

Supplementary Figure 7. Docking prediction of IP3K-ligands. a, GOLD solutions for compounds **7b-s**, **8** and **9** bound in IP3K active site that show how phosphates mimic $InsP_3$ phosphate positions. The color code is the same as Fig. 3. The plots from Mogul[3] display the distribution of the corresponding torsion angles within structures containing similar fragments in CSD; the red lines mark the values of the torsion angles from the docked molecules. The colored arrows refer to specific bonds in the protein pictures and the histograms. In these three cases the conformations of the ligands present non-favored torsions. **b,** GOLD best 10 solutions for designed compounds **10** (L-*chiro*-inositol 1,4,6-trisphosphate) and **11** (3-deoxy-3-hydroxymethyl-*myo*-inositol 1,4,5-trisphosphate) using ensemble docking. Compound **10** could display two energetically similar conformations (chair 1 (left) and chair 2 (middle)). The color code is the same as Supplementary Fig. 6a. Docking suggests that neither one of these conformations would bind the IP3K active site. In contrast, compound **11** is predicted to bind in a similar way to compound **5-s. c,** A selected solution from docking results with compound **11** suggests that an aberrant phosphorylation of a primary hydroxyl resting on a *myo*-inositol ring would be possible since the distance between the reactive centers depicted seems compatible with catalysis.

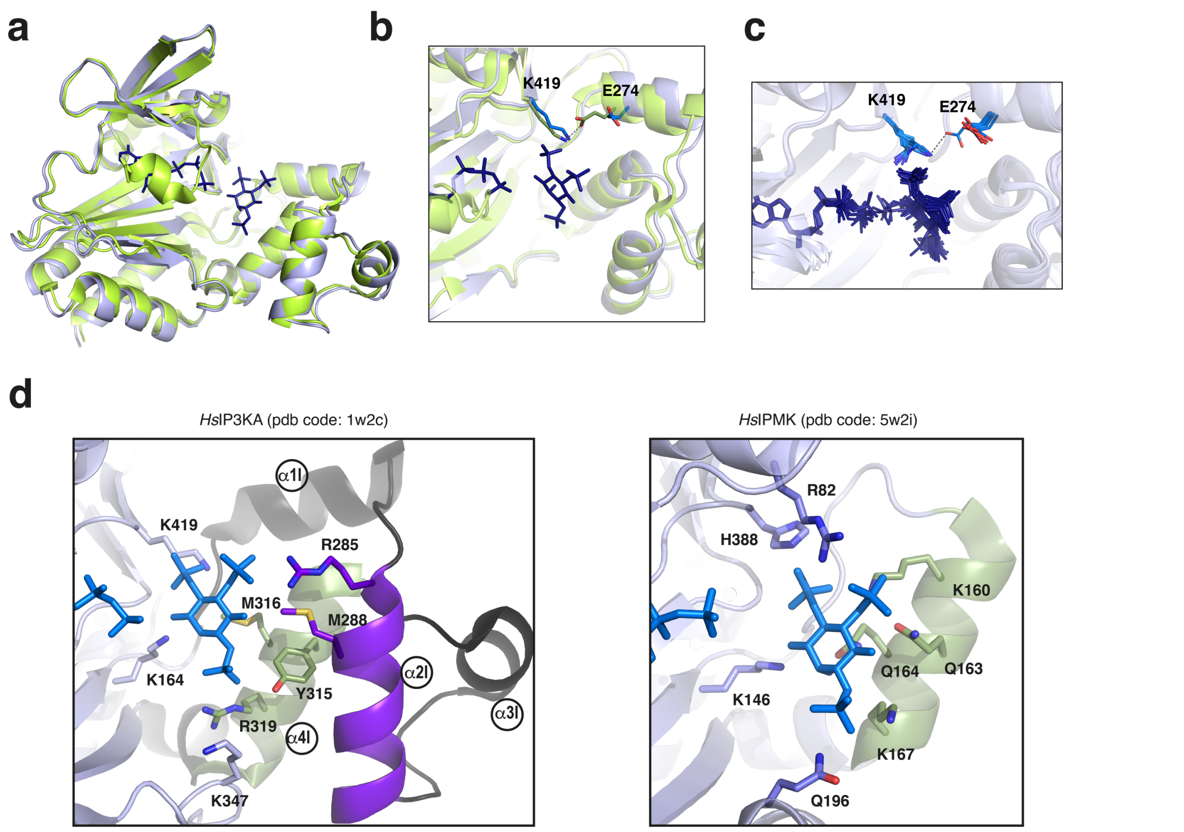

Supplementary Figure 8. IP-lobe changes in IP3K-KD and comparison with IPMK. a, Crystal structure superposition between apo IP3K-KD (green, code 1w2f) and IP3K in complex with the substrate InsP$_3$ and AMP-PNP (blue, code 1w2c). The superposition shows an overall shrinking of the structure in the absence of ligands noticeable in the IP-lobe and other InsP proximal regions. **b,** A zoom of the InsP$_3$ binding region showing a hydrogen bond in the IP3K structure that is broken upon substrate binding which, together with the protein-ligand interactions formed, provokes an opening of the ligand cavity. Note that the IP-lobe moves as a rigid body upon substrate binding whereas the local plasticity involved in ligand discrimination shown in this work (Fig. 5) is predominantly restricted to $\alpha 2_I$. **c,** Superposition of structures obtained in this work showing, as expected, that the mentioned hydrogen bond is not formed upon ligand binding. In just one case, the IP3K complex with 6b-s, we observe some electron density corresponding with Glu274 being oriented to form the bond. As this complex does not present good binding to IP3K, we assume that the ligand occupancy is not high, suggesting that we could have mixed apo- and ligand-bound IP3K conformations. **d,** IP-lobe comparison between IP3KA (left) and IPMK (right) structures. Both structures are shown in cartoon representation highlighting the InsP (blue) and the InsP binding residues as sticks, and the IP lobe helices in different colors (IP3K: $\alpha 1_I$ and $\alpha 3_I$:black, $\alpha 2_I$ purple and $\alpha 4_I$:green; IPMK: green, equivalent to IP3K $\alpha 4_I$)

Supplementary Tables

Supplementary Table 1. Data collection and refinement statistics of IP3K complexes structures.

	**1-s**	**1-p**	**2-s**	**3-s**
Nucleotide, ion	AMP-PNP, Mn	ADP, Mn	AMP-PNP, Mn	ATP, Mn
Accession code	8PP8	8PP9	8PPA	8PPB
**Data collection**				
Beamline	XALOC (ALBA)	XALOC (ALBA)	XALOC (ALBA)	XALOC (ALBA)
Temperature (K)	100	100	100	100
Wavelength (Å)	0.999871	0.999871	0.979264	0.979264
Space group	$C\ 2\ 2\ 2_1$	$C\ 2\ 2\ 2_1$	$C\ 2\ 2\ 2_1$	$C\ 2\ 2\ 2_1$
Cell dimensions				
a, *b*, *c* (Å)	72.56, 97.35, 191.18	72.50, 97.51, 191.49	72.65, 97.92, 192.07	71.77, 97.69, 191.94
α, β, γ (°)	90.00, 90.00, 90.00	90.00, 90.00, 90.00	90.00, 90.00, 90.00	90.00, 90.00, 90.00
Solvent (%)	54.27	58.38	54.80	54.11
Molecules per ASU	2	2	2	2
Resolution (Å)	95.59 – 1.59 (1.62 – 1.59)	58.18 – 1.73 (1.76 – 1.73)	49.86 – 1.73 (1.76 – 1.73)	49.54 – 1.80 (1.84 – 1.80)
Total reflections	829,294 (22,087)	688,335 (29,061)	755,933 (47,115)	720,102 (33,698)
Unique reflections	90,163 (4,163)	70,868 (3,453)	64,275 (3,876)	62,723 (3,648)
R_{pim}	0.015 (0.447)	0.021 (0.439)	0.037 (0.417)	0.028 (0.402)
$I / \sigma (I)$	21.8 (1.2)	17.5 (1.4)	12.1 (1.8)	15.1 (1.6)
Completeness (%)	99.5 (93.5)	100.0 (99.9)	89.5 (100.0)	99.8 (98.3)
Redundancy	9.2 (5.3)	9.7 (8.4)	11.8 (12.2)	11.5 (9.2)
CC1/2	0.999 (0.590)	0.999 (0.627)	0.994 (0.756)	0.998 (0.679)
Wilson B-factor ($Å^2$)	29.700	32.400	27.847	28.529
**Refinement**				
R_{work} / R_{free}	0.193 / 0.212	0.194 / 0.210	0.193 / 0.218	0.195 / 0.216
No. atoms (chain A)				
Protein	2,183	2,203	2,282	2,237
Inositide	24	28	24	23
Nucleotide	31	27	31	31
Mn^{2+} / Mg^{2+} ions	1	1	2	2
B-factors (chain A)				
Protein	37.02	39.79	38.65	39.60
Inositide	35.65	40.78	42.61	47.62
Nucleotide	39.97	41.98	38.62	33.34
Mn^{2+} / Mg^{2+} ions	42.64	51.56	54.68	43.63
Residues (chain A)	270	271	275	276
Ramachandran plot				
Favoured (%)	99	98	99	98
Outliers (%)	0	0	0	0
R.m.s. deviations				
Bond lengths (Å)	0.0059	0.0068	0.0064	0.0073
Bond angles (°)	1.3785	1.4333	1.4016	1.4468

* *Statistics for the highest-resolution shell are shown in parentheses.*

(continued to next page)

	**4-s**	**5-s**	**5-p**	**6a-s**
Nucleotide, ion	ATP, Mn	ATP, Mn	ADP, Mn	AMP-PNP, Mg
Accession code	8PPC	8PPD	8PPE	8PPF
**Data collection**				
Beamline	XALOC (ALBA)	XALOC (ALBA)	XALOC (ALBA)	XALOC (ALBA)
Temperature (K)	100	100	100	100
Wavelength (Å)	0.979264	0.979178	0.979185	0.979264
Space group	$C\,2\,2\,2_1$	$C\,2\,2\,2_1$	$C\,2\,2\,2_1$	$C\,2\,2\,2_1$
Cell dimensions				
a, b, c (Å)	71.92, 97.90, 191.88	72.16, 97.52, 192.06	72.55, 97.55, 192.06	72.82, 97.59, 191.93
α, β, γ (°)	90.00, 90.00, 90.00	90.00, 90.00, 90.00	90.00, 90.00, 90.00	90.00, 90.00, 90.00
Solvent (%)	54.29	54.31	54.18	54.72
Molecules per ASU	2	2	2	2
Resolution (Å)	49.66 – 1.92 (1.97 – 1.92)	48.01 – 1.77 (1.81 – 1.77)	58.28 – 1.59 (1.66 – 1.59)	48.84 – 1.85 (1.89 – 1.85)
Total reflections	651,450 (27,442)	724,352 (36,105)	1,058,343 (44,566)	595,934 (18,772)
Unique reflections	51,230 (2,960)	66,119 (3,675)	80,256 (4,014)	56,714 (2,593)
R_{pim}	0.032 (0.299)	0.024 (0.427)	0.014 (0.497)	0.004 (0.263)
$I / \sigma (I)$	13.3 (1.8)	16.0 (1.6)	22.8 (1.3)	12.0 (2.2)
Completeness (%)	98.4 (84.8)	99.7 (98.0)	*sph.* 88.2 (34.9) *ellip.* 95.4 (64.8)	96.8 (74.3)
Redundancy	10.0 (7.6)	11.0 (9.8)	13.2 (11.1)	10.5 (7.2)
CC1/2	0.997 (0.799)	0.998 (0.607)	1.000 (0.566)	0.994 (0.779)
Wilson B-factor (Å^2)	31.616	30.204	29.000	29.545
**Refinement**				
R_{work} / R_{free}	0.193 / 0.219	0.202 / 0.231	0.200 / 0.240	0.196 / 0.228
No. atoms (chain A)				
Protein	2,235	2,177	2,203	2,188
Inositide	24	25	29	24
Nucleotide	31	31	27	31
Mn^{2+} / Mg^{2+} ions	2	1	1	0
B-factors (chain A)				
Protein	43.49	40.44	37.59	41.01
Inositide	52.56	41.56	75.44	49.46
Nucleotide	38.81	39.23	41.56	42.83
Mn^{2+} / Mg^{2+} ions	40.65	46.69	35.07	0.00
Residues (chain A)	276	270	272	270
Ramachandran plot				
Favoured (%)	97	98	99	98
Outliers (%)	0	0	0	0
R.m.s. deviations				
Bond lengths (Å)	0.0079	0.0058	0.0057	0.0050
Bond angles (°)	1.4997	1.3579	1.3145	1.3556

* *Statistics for the highest-resolution shell are shown in parentheses.*

(continued to next page)

	6a-p	6b-s	7a-s	7a-p
Nucleotide, ion	ADP, Mn	ATP, Mn	ATP, Mn	ADP, Mn
Accession code	8PPG	8PPH	8PPI	8PPJ
**Data collection**				
Beamline	ID23-1 (ESRF)	ID23-1 (ESRF)	ID23-2 (ESRF)	ID23-1 (ESRF)
Temperature (K)	100	100	100	100
Wavelength (Å)	0.885600	0.774899	0.873128	0.885600
Space group	$C\ 2\ 2\ 2_1$	$C\ 2\ 2\ 2_1$	$C\ 2\ 2\ 2_1$	$C\ 2\ 2\ 2_1$
Cell dimensions				
a, *b*, *c* (Å)	71.28, 97.10, 190.76	72.05, 97.50,190.88	73.95, 98.12, 193.16	72.53, 97.06, 191.13
α, β, γ (°)	90.00, 90.00, 90.00	90.00, 90.00, 90.00	90.00, 90.00, 90.00	90.00, 90.00, 90.00
Solvent (%)	53.23	53.94	55.94	54.10
Molecules per ASU	2	2	2	2
Resolution (Å)	190.76 – 1.75 (1.78 – 1.75)	48.75 – 1.70 (1.73 – 1.70)	193.16 – 1.65 (1.68 – 1.65)	191.13 – 1.75 (1.78 – 1.75)
Total reflections	885,987 (49,977)	995,471 (54,823)	999,825 (47,890)	925,614 (53,360)
Unique reflections	67,053 (3,682)	74,155 (3,910)	77,850 (4,142)	68,270 (3,749)
R_{pim}	0.024 (0.572)	0.017 (0.453)	0.024 (0.493)	0.023 (0.467)
$I / \sigma (I)$	13.6 (1.3)	18.9 (1.7)	15.5 (1.7)	16.6 (1.6)
Completeness (%)	100.0 (100.0)	100.0 (100.0)	91.9 (100.0)	99.9 (99.9)
Redundancy	13.2 (13.6)	13.4 (14.0)	12.8 (11.6)	13.6 (14.2)
CC1/2	0.999 (0.676)	0.999 (0.663)	0.999 (0.620)	0.999 (0.661)
Wilson B-factor (Å^2)	34.625	33.244	25.115	29.400
**Refinement**				
R_{work} / R_{free}	0.206 / 0.243	0.198 / 0.231	0.185 / 0.214	0.197 / 0.234
No. atoms (chain A)				
Protein	2,213	2,241	2,230	2,234
Inositide	28	24	25	29
Nucleotide	27	31	31	27
Mn^{2+} / Mg^{2+} ions	2	2	1	3
B-factors (chain A)				
Protein	47.17	45.75	34.34	40.76
Inositide	56.41	72.57	38.07	45.18
Nucleotide	43.41	44.28	36.18	32.71
Mn^{2+} / Mg^{2+} ions	68.90	51.91	54.74	72.79
Residues (chain A)	274	275	275	275
Ramachandran plot				
Favoured (%)	97	98	98	99
Outliers (%)	0	0	0	0
R.m.s. deviations				
Bond lengths (Å)	0.0068	0.0067	0.0051	0.0072
Bond angles (°)	1.4419	1.3876	1.3793	1.3483

* *Statistics for the highest-resolution shell are shown in parentheses.*

Supplementary Table 2. IC_{50} and inferred K_d values for selected IP3K ligands obtained from 2-FITC-$InsP_3$ displacement curves.

Compound	IC_{50} (nM)*	Confidence Interval	K_d (nM)	Confidence Interval**
**$InsP_3$**	140	(134-146)	136	130-142
**$InsP_4$**	60	(58-62)	58	56-60
**1-s**	36	(35-37)	35	34-36
**1-p**	51	(50-53)	50	49-51
**5-s**	972	(914-1,033)	944	888-1003
**5-p**	875	(826-927)	850	802-900
**6a-s**	205	(195-215)	199	189-210
**6b-s**	8,977	(7,794-10,340)	8,719	7,570-10,043
**7a-s**	656	(611-705)	637	593-685
**7b-s**	6,986	(6,282-7,768)	6,785	6,102-7,545

**mean value obtained from n=4 replicates*
***obtained by simple linear approximation*

Supplementary Table 3. Temperature data T_i (and ΔT_i) of thermal shift assays of apo IP3K-KD and in complex with the ligands.

Complex	Average T_i (ΔT_i) (°C)	
	Transition 1	Transition 2
IP3K-KD (apo)	44.4 ± 0.4 (*Reference*)*	53.8 ± 0.2 (*Reference*)*
IP3K-KD/$InsP_3$	52.9 ± 0.2 (8.5 ± 0.3)	58.2 ± 0.2 (4.4 ± 0.2)
IP3K-KD/$InsP_4$	53.5 ± 0.2 (9.1 ± 0.4)	58.8 ± 0.2 (5.0 ± 0.3)
**$InsP_3$ isomers**		
IP3K-KD/**1-s**	55.6 ± 0.1 (11.2 ± 0.3)	60.7 ± 0.2 (6.9 ± 0.2)
IP3K-KD/**1-p**	54.7 ± 0.2 (10.3 ± 0.3)	59.9 ± 0.2 (6.1 ± 0.2)
IP3K-KD/**2-s**	53.1 ± 0.3 (8.7 ± 0.4)	58.4 ± 0.2 (4.6 ± 0.2)
IP3K-KD/**3-s**	52.0 ± 0.2 (7.6 ± 0.3)	57.4 ± 0.1 (3.6 ± 0.2)
IP3K-KD/**4-s**	51.7 ± 0.1 (7.3 ± 0.3)	57.3 ± 0.1 (3.5 ± 0.2)
**3-substituted analogues**		
IP3K-KD/**5-s**	51.3 ± 0.2 (6.9 ± 0.4)	57.0 ± 0.1 (3.2 ± 0.2)
IP3K-KD/**5-p**	52.1 ± 0.1 (7.7 ± 0.3)	57.6 ± 0.1 (3.8 ± 0.2)
IP3K-KD/**6a-s**	51.5 ± 0.2 (7.1 ± 0.3)	57.3 ± 0.1 (3.5 ± 0.2)
IP3K-KD/**6b-s**	45.7 ± 0.5 (1.3 ± 0.6)	54.6 ± 0.0 (0.8 ± 0.2)
IP3K-KD/**7a-s**	49.7 ± 0.4 (5.3 ± 0.5)	56.1 ± 0.3 (2.3 ± 0.3)
IP3K-KD/**7b-s**	45.6 ± 0.7 (1.2 ± 0.7)	54.4 ± 0.3 (0.6 ± 0.3)
**Ribophostin-like**		
IP3K-KD/**8**	44.2 ± 0.2 (-0.2 ± 0.3)	54.1 ± 0.1 (0.3 ± 0.2)
IP3K-KD/**9**	44.5 ± 0.4 (0.1 ± 0.5)	54.5 ± 0.1 (0.7 ± 0.2)

**The ΔT_i has been calculated assuming T_i of* IP3K-KD *(apo) as reference for each transition value*

Supplementary References

1. Riley, A. M., Guédat, P., Schlewer, G., Spiess, B. & Potter, B. V. L. Conformationally Restricted Cyclic Phosphate Analogue of Inositol Trisphosphate: Synthesis and Physicochemical Properties. *J. Org. Chem.* **63**, 295–305 (1998).

2. Whitfield, H. *et al.* Allosteric Site on SHIP2 Identified Through Fluorescent Ligand Screening and Crystallography: A Potential New Target for Intervention. *J. Med. Chem.* **64**, 3813–3826 (2021).

3. Bruno, I. J. *et al.* Retrieval of Crystallographically-Derived Molecular Geometry Information. *J. Chem. Inf. Comput. Sci.* **44**, 2133–2144 (2004).
