# Supplementary material for: Substrate promiscuity of inositol 1,4,5-trisphosphate kinase driven by structurally-modified ligands and active site plasticity
Source: Nat Commun. 2024 Feb 19;15:1502. doi: 10.1038/s41467-024-45917-5 (PMC10876669; doi:10.1038/s41467-024-45917-5)
Supplement: Supplementary file 3 — Description of Additional Supplementary files [file 41467_2024_45917_MOESM3_ESM.pdf]

Description of Additional Supplementary Information

File name: Supplementary Movie 1
Description: Motion observed in a2I helix in IP3K upon different ligands binding.
